# The Evolving Landscape of the Medicine-Pediatrics Workforce: Lessons From the Last 10 Years

**DOI:** 10.7759/cureus.78869

**Published:** 2025-02-11

**Authors:** Kristin Wong, Nabil Baker, Natalie Sous, Ravi Upadhyay, Nicole Reynoso-Vasquez, David Cennimo, Megna Khatri, Alan Tso, Luis Alzate-Duque, Jon Sicat, Brent Parris, Jayne Barr

**Affiliations:** 1 Internal Medicine-Pediatrics, Rutgers University New Jersey Medical School, Newark, USA; 2 Internal Medicine-Pediatrics, University of Chicago Medicine, Chicago, USA; 3 Infectious Disease, Veterans Affairs Medical Center, East Orange, USA; 4 Internal Medicine-Pediatrics, Newark Beth Israel Medical Center, Newark, USA; 5 Internal Medicine-Pediatrics, MetroHealth Medical Center, Cleveland, USA

**Keywords:** hospital medicine, med-peds careers, med-peds workforce, primary care, workforce survey

## Abstract

Objective

As a combined subspecialty, internal medicine-pediatrics (Med-Peds) physicians have played a significant role in both primary care and subspecialty care across the country. Over the last decade, the workforce has continued to grow and evolve. Thus, this study aims to characterize the current landscape of the Med-Peds workforce to address preconceptions and shine a light on current practice characteristics and career paths. Understanding factors influencing this group’s career path is vital in addressing the needs of an aging population and physician shortages.

Methods

The survey study was designed by the Committee on Pediatric Workforce of the American Academy of Pediatrics (AAP) and modified by the Section of Medicine-Pediatrics (SOMP) Executive Committee. The survey was distributed electronically via SurveyMonkey to 3,536 AAP Section members and 3,230 Med-Peds members of the American Medical Association (AMA). There were 1,395 respondents, 956 of whom had completed Med-Peds residency training by 2022 and were eligible to be included in this study. Descriptive statistics and analysis, including frequency distributions and measures of central tendency, were used to summarize all responses. Statistical tests such as t-tests and z-proportions were used for comparative analysis.

Results

The majority of the Med-Peds physician workforce continued to practice across all ages (89.6%) as primary care physicians (65.4%) and worked an average of 50 hours per week. However, there has been a growing number of hospitalists (27.4%) and a trend toward practices in urban communities at academic medical centers. Growing financial concerns about educational debt and pay gaps between internal medicine and pediatrics as well as differences between early and late-career physicians also revealed changes in career choices, but overall satisfaction in training and specialty decisions was maintained.

Conclusions

Despite a multitude of external pressures affecting the workforce, Med-Peds physicians were satisfied with their training. They continue to add to the primary care sector and continue to see patients of all ages. While this versatile workforce can aid in the provision of care to populations particularly vulnerable during their transitions of care, such as children with complex medical needs, factors like increasing educational debt, widening pay gaps, and local competition will contribute to changes seen in work type and career paths. Further research to understand the career decisions of this workforce is needed to better address the rising physician shortages plaguing the entire country.

## Introduction

As a combined subspecialty, internal medicine-pediatrics (Med-Peds) physicians continue to play a significant role in both primary care and subspecialty care across the country. Approved by the American Board of Internal Medicine (ABIM) and American Board of Pediatrics (ABP) in 1967, combined Med-Peds residencies have grown to over 77 training programs and have over 1,500 active residents [1], with almost 400 graduates each year. In 2013, the American Academy of Pediatrics (AAP) research team and leadership from the Section of Medicine-Pediatrics (SOMP) developed the first comprehensive national survey of the Med-Peds workforce [2]. The survey revealed that Med-Peds physicians maintain both boards and practice medicine across the age range. As such, Med-Peds graduates continue to add to primary care, hospital medicine, and subspecialty care workforces, along the continuum of practice settings across the country.

Due to an aging population as well as current and projected workforce shortages, concerns about the ability of the healthcare workforce to meet the demand, particularly in primary care, have been emphasized. The American Academy of Medical Colleges (AAMC) projected a shortage of 20,200-40,400 primary care providers in the United States (US) and 3,800-13,400 medical subspecialists by 2036 [3]. Additionally, between 2008 and 2017, 10.4% of adult primary care physicians exited practice [4], and AAMC has speculated that more than a third of currently active physicians could retire in the next decade based on a traditional retirement age of 65 or older [3]. Further exacerbating these projections, 4.7% of internal medicine residency positions and 8% of pediatric residency positions remained unfilled in the 2024 National Residency Match [5]. Med-Peds resident positions accounted for 3.6% of medicine (390 of 10,681) and 12.4% of pediatric positions (390 of 3,139) and were completely filled [5]. In 2021, there were 5,701 active Med-Peds physicians with activities ranging from patient care, teaching, research, and administration [6]. In light of these mounting demands, it is important to understand trends within the Med-Peds workforce and recognize their flexibility in the scope of practice inherent to combined training and role in both primary and subspecialty care. Healthcare systems that recognize the strengths of this workforce can create flexible staffing models to care for an evolving spectrum of patients. This study aims to review the practices, perceptions, and careers of the current Med-Peds workforce.

## Materials and methods

Study design

The Survey Initiative, organized by the Committee on Pediatric Workforce of the AAP, develops periodic surveys distributed to physician members as a means of reviewing and analyzing its workforce. This questionnaire was modified by the SOMP’s Executive Committee and approved by the AAP’s research team. The final survey consisted of 105 questions covering a core set of variables and specialty-specific questions including demographics, practice characteristics, practice-specific questions, and career planning and satisfaction. Within the survey tool, display and skip logic were used to direct respondents to applicable question sets. Single-answer, multiple-answer, constant sum, and free-text questions were used. The survey was approved and the study was deemed exempt by the AAP Institutional Review Board.

Inclusion and exclusion criteria

**Figure 1:**
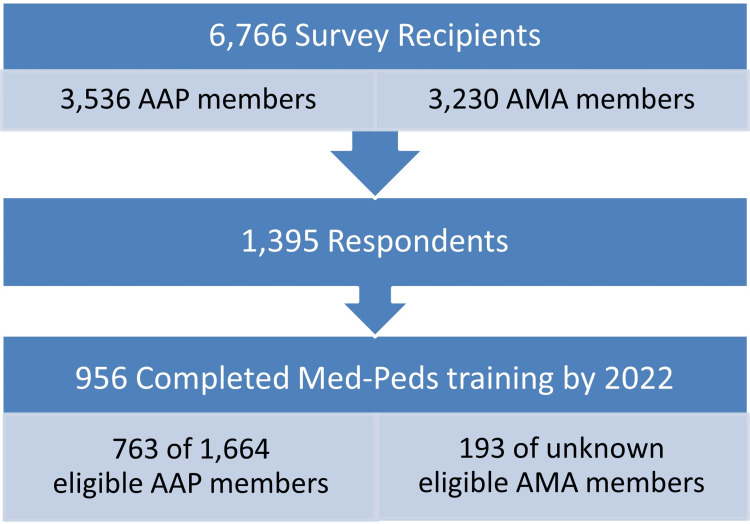
Participant flow diagram AAP: American Academy of Pediatrics; AMA: American Medical Association; Med-Peds: Internal medicine-pediatrics

A total of 3,536 Med-Peds physicians were identified from the AAP’s member listserv. Med-Peds physicians from the American Medical Association’s (AMA) Medical Marketing Service (MMS) listserv were also identified and compared with the AAP’s list. Duplicates were removed and an additional 3,230 AMA members were added to the list for a total of 6,766 recipients. Only survey responses indicating “Yes” to having completed Med-Peds residency training by 2022 or earlier were included in this study (Figure 1). 

Data collection

The survey was distributed through email with an electronic link via SurveyMonkey, an online survey platform. Recipients received quarterly reminders from December 3, 2022, to June 19, 2023. Specific data concerning demographics, practice characteristics, practice-specific questions, and career planning and satisfaction were extracted and analyzed for this study. 

Statistical analysis

Descriptive statistics and analysis, including frequency distributions and measures of central tendency, were used to summarize all responses. Additional statistical tests such as t-tests and z-proportions were used for comparative analysis. Results were compared with the published results obtained from the AAP's Med-Peds workforce survey done in 2013 [2].

## Results

**Figure 2:**
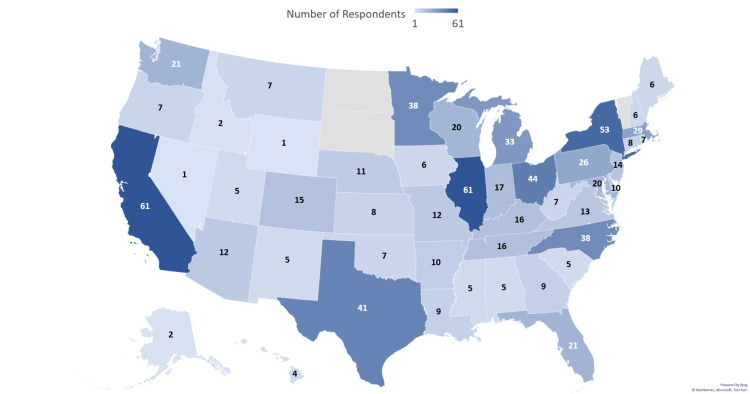
Number of Med-Peds respondents by state Med-Peds: Internal medicine-pediatrics Credits: Kristin Wong

There were 1,395 respondents, of which 956 were considered eligible as having completed Med-Peds residency training. Of those, 763 of 1,664 eligible respondents were from the AAP listserv, resulting in a response rate of 45.8%. A total of 193 respondents were eligible physicians from the AMA MMS listserv, but we could not determine the total number of eligible AMA members and, therefore, the response rate (Figure 1). A total of 802 respondents completed the entirety of the survey, while others left some survey questions blank, resulting in a variable denominator within the responses. Due to skip and display logic questions, there was also variability in the responses to certain question sets depending on whether the questions applied to the respondent. Most respondents were members of the AAP (705/730, 96.6%), represented a wide range of geographical practice locations across the US (Figure 2), and self-identified as heterosexual, non-Hispanic, White women (337/599, 56.3%).

About 885 of 942 (94%) held allopathic doctorates, 46 (4.9%) held osteopathic doctorates, and 11 (1.2%) indicated an “other” type of medical degree. Most respondents had completed medical school in the US (778/802, 97%) and were currently dual-boarded (834/942, 88.4%). About 206 of 938 (22%) Med-Peds graduates had completed subspecialty or fellowship training (Table 1).

**Table 1:** Characteristics of Med-Peds physicians The numbers do not sum to 100% due to rounding and not all respondents answered every question resulting in variable denominators per question. Med-Peds: Internal medicine-pediatrics; ABP: American Board of Pediatrics; ABIM: American Board of Internal Medicine; DO: Doctor of Osteopathic Medicine; MD: Doctor of Medicine; AAP: American Academy of Pediatrics

Physician characteristics	n	%
Gender identity		
Man	311/802	38.80%
Woman	478/802	59.60%
Non-binary	3/802	0.40%
Self-described	0/802	0.00%
Declined	10/802	1.20%
Race		
American Indian or Alaskan Native	8/796	1.00%
Asian	116/796	14.60%
Black/African American	35/796	4.40%
Middle Eastern/North African	7/796	0.90%
Native Hawaiian/Pacific Islander	1/796	0.10%
White	609/796	76.50%
Other	8/796	1.00%
Declined to respond	33/796	4.10%
Ethnicity		
Hispanic or Latinx	19/798	2.40%
Non-Hispanic White	762/798	95.50%
Declined to respond	17/798	2.10%
Sexual identity		
Bisexual	14/794	1.80%
Lesbian/gay	28/794	3.50%
Straight	716/794	90.20%
Other	2/794	0.30%
I don't know	2/794	0.30%
Declined to respond	32/794	4.00%
Medical school location		
United States	778/802	97.00%
Canada	0/802	0.00%
Caribbean	8/802	1.00%
Other	16/802	2.00%
Professional degree		
MD	885/942	93.90%
DO	46/942	4.90%
Other	11/942	1.20%
Current board certification		
ABP only	37/942	3.90%
ABIM only	44/942	4.70%
Dual certification	834/942	88.40%
Past certified	20/942	2.10%
Never certified	7/942	0.70%
Subspecialty/fellowship training	206/938	22.00%
Organization membership		
AAP	705/730	96.60%
American College of Physicians	580/730	79.50%
AAP's Section on Medicine-Pediatrics	478/730	65.50%
Society for Hospital Medicine	106/730	14.50%
Another organization or AAP section	134/730	18.40%
Years since residency graduation		
Mean	15.4 years	
Range	1 to 43 years	

Practice and academic characteristics

Most of the respondents identified as primary care physicians (491/751, 65.4%) and practiced in a variety of settings including medical schools/hospitals (318/872, 36.5%) or family/Med-Peds group practices (145/872, 16.6%). Most respondents worked in urban communities (428/861, 49.7%), particularly as hospitalists (136/206, 66%) or subspecialists (99/140, 70.7%). A large proportion of respondents held faculty appointments (519, 59.5%) and pursued scholarly activities including quality assessment and quality improvement (QA/QI) (312/753, 41.4%) and medical education research (161/753, 21.4%). On average, respondents worked 50 hours/week for a full-time position (724/863, 83.9% of respondents worked full-time) and 32 hours/week for a part-time position (139/863, 16.1% of respondents worked part-time), with an average of 8.6 hours/week spent on documentation (n=853) and 70.1% time dedicated to direct patient care (807/840, 96.1% of respondents provided direct patient care). Of those providing direct patient care, 638 of 712 (89.6%) saw patients in both adult (≥18 years of age) and pediatric (<18 years of age) age groups and provided care evenly across the age range (Tables 2-3).

**Table 2:** Practice characteristics of Med-Peds physicians The numbers do not sum to 100% due to rounding and not all respondents answered every question resulting in variable denominators per question. ᵃ: Of those providing that type of work, the average % of FTE spent; FTE: Full-time effort; PCP: Primary care physicians; HMO: Health Maintenance Organization

Practice characteristics	n	%	Avg % FTEᵃ
Practice			
PCP	491/751	65.40%	
Hospitalist	206/751	27.40%	
Subspecialist	141/751	18.80%	
Practice setting			
Medical school/hospital (or parent university)	318/872	36.50%	
Family or Med-Peds group practice	145/872	16.60%	
Multi-specialty group	96/872	11.00%	
Community/staff model hospital	83/872	9.50%	
Non-profit community health center or health department	63/872	7.20%	
Solo practice	25/872	2.90%	
Specialty group practice	15/872	1.70%	
HMO staff/group model	11/872	1.30%	
Pediatric group practice	9/872	1.00%	
Uniformed Health Services clinic	0/872	0.00%	
Other (please specify)	107/872	12.30%	
Primary practice locale			
Urban	428/861	49.70%	
Suburban	316/861	36.70%	
Rural	117/861	13.60%	
Primary role			
Employee	696/786	88.50%	
Full or part-owner	66/786	8.40%	
Independent contractor	28/786	3.60%	
Other (please specify)	9/786	1.10%	
Type of work			
Direct patient care	807/840	96.10%	70.13%
Administration	529/840	63.00%	24.11%
Teaching	528/840	62.90%	13.53%
Clinical research	116/840	13.80%	14.24%
Basic science research	8/840	1.00%	24.37%
Health services research	56/840	6.70%	21.55%
Medical activities not involving the direct care of patients (e.g., committee work, consulting with agencies)	292/840	34.80%	10.14%
Other	45/840	5.40%	33.11%
Patient care work			
Primary care pediatrics	558/798	69.90%	43.18%
Pediatric medical subspecialty	200/798	25.10%	58.22%
Pediatric surgical specialty	3/798	0.40%	35.00%
Another specialty, including adult	648/798	81.20%	67.84%
Patients cared for by age distribution			
2 years or younger	612/712	86.00%	16.90%
3-17 years	649/712	91.20%	19.80%
18-25 years	677/712	95.10%	12.90%
26-40 years	640/712	89.90%	15.70%
41-64 years	631/712	88.60%	22.10%
65+ years	621/712	87.20%	24.70%

**Table 3:** Academics of Med-Peds physicians The numbers do not sum to 100% due to rounding and not all respondents answered every question resulting in variable denominators per question. QA/QI: Quality assessment and quality improvement

Academic details	n	%
Academic appointment		
None	322/872	36.90%
Non-tenured	445/872	51.00%
Tenured	74/872	8.50%
Other	31/872	3.60%
Academic rank	518/872	59.40%
Instructor	55/518	10.60%
Assistant professor	243/518	46.90%
Associate professor	125/518	24.10%
Full professor	45/518	8.70%
Adjunct	33/518	6.40%
Other	17/518	3.30%
Types of scholarship		
None	310/753	41.20%
QA/QI	312/753	41.40%
Medical education research	161/753	21.40%
Clinical research	138/753	18.30%
Health service/population health research	129/753	17.10%
Bench research	7/753	0.90%
Other	16/753	2.10%
Work with other providers		
Hospitalists	341/742	46.00%
Nurse practitioners	547/742	73.70%
Physician assistants	358/742	48.20%
Fellows	185/742	24.90%
Residents	451/742	60.80%
Medical students	436/742	58.80%
Other students (e.g., nurse practitioner, physician assistant, nursing, undergraduate)	210/742	28.30%

Financial and career attitudes

Most Med-Peds physicians reported working as employees and received benefits such as malpractice and health insurance, reimbursement for professional expenses, retirement, disability, and work bonuses. However, few received tuition (126/775, 16.3%) or loan reimbursements (86/775, 11.1%). While there were several Med-Peds physicians without debt (426/787, 54.1%), 200 of 787 (25.4%) respondents were somewhat concerned or very concerned about debt. This discrepancy was more evident when comparing recent graduates (early-career) and graduates more than 10 years out (late-career) (Tables 4-5). Over 50% of Med-Peds physicians’ compensation was dependent on relative value units (RVUs) (459/788, 58.2%) and less than 20% felt that Med-Peds had a negative effect on salary (132/755, 17.5%). Of note, many respondents believed their salary would be higher if employed solely in medicine (305/754, 40.5%) versus pediatrics (10/751, 1.3%). Respondents also believed that the group they worked for saw advantages to combined Med-Peds training (394/756, 52.1%) (Table 4).

**Table 4:** Compensation and salary perceptions of Med-Peds physicians The numbers do not sum to 100% due to rounding and not all respondents answered every question resulting in variable denominators per question. N/A: Not applicable; RVUs: Relative value units

Compensation and salary details	n	%
Commonly received compensation packages		
Malpractice insurance	718/775	92.60%
Health insurance	711/775	91.70%
Reimbursement for professional expenses such as licensure, dues, or meeting expenses	662/775	85.40%
Retirement	612/775	79.00%
Life insurance	597/775	77.00%
Short-term disability	528/775	68.10%
Long-term disability	511/775	65.90%
Bonus	462/775	59.60%
Paid family medical leave	361/775	46.60%
Dependent care coverage	285/775	36.80%
Paid family care leave	258/775	33.30%
Tuition reimbursement	126/775	16.30%
Loan repayment	86/775	11.10%
Other	33/775	4.30%
Compensation dependent on RVUs	459/788	58.20%
Concerned about educational debt		
Very concerned	82/787	10.40%
Somewhat concerned	118/787	15.00%
Not concerned	161/787	20.50%
N/A - no debt	426/787	54.10%
Effect on salary		
Positive	265/755	35.10%
Neutral	358/755	47.40%
Negative	132/755	17.50%
True statements		
My salary would be higher if hired by medicine	305/754	40.50%
My salary would be higher if hired by pediatrics	10/751	1.30%
There is competition with local internists	100/751	13.30%
There is competition with local pediatricians	214/751	28.50%
I am responsible for covering my own practice costs	75/751	10.00%
The group that I work for sees advantages to Med-Peds training over categorical training	394/756	52.10%
I am completely grant-funded	9/749	1.20%

**Table 5:** Significant differences between early versus late-career Med-Peds physicians The numbers do not sum to 100% due to rounding and not all respondents answered every question resulting in variable denominators per question. ^†^: Early-career (<10 years after graduation); ^‡^: Late-career (>10 years after graduation); PCP: Primary care physicians; ABP: American Board of Pediatrics

Variables	<10 years^†^		>10 years^‡^		Two-tailed z-scores	p-values
	n	%	n	%		
Demographics						
ABP-only certified	5/346	1.40%	32/590	5.40%	-3.016	0.0025
Dual medicine and pediatrics certified	317/346	91.60%	511/590	86.60%	2.315	0.0203
Identify as Asian	60/288	20.80%	56/504	11.10%	3.723	0.0002
Identify as White	202/288	70.10%	403/504	80.00%	-3.131	0.0017
Members of AAP's Section on Medicine-Pediatrics	195/254	76.80%	282/473	59.60%	4.642	<0.00001
Members of Society for Hospital Medicine	63/254	24.80%	43/473	9.10%	5.7234	<0.00001
Members of American College of Physicians	215/254	84.60%	362/473	76.50%	2.5773	0.00988
Practice characteristics						
Work as PCPs	147/258	57.00%	341/490	69.60%	-3.444	0.0006
Work as hospitalists	112/258	43.40%	94/490	19.20%	7.051	<0.00001
Dual-practicing PCPs and hospitalists	26/258	10.10%	28/490	5.70%	2.192	0.0285
Work in solo practices	1/302	0.30%	24/566	4.20%	-3.28	0.001
Work in multi-specialty group practices	20/302	6.60%	76/566	13.40%	-3.045	0.0024
Work at medical schools/hospitals	142/302	47.00%	174/566	30.70%	4.747	<0.00001
Work in urban communities	174/294	59.20%	254/563	45.10%	3.91	0.0001
Work part-time	28/294	9.50%	110/565	19.50%	-3.766	0.0002
Academics						
Tenured academic appointment	37/302	12.30%	37/566	6.50%	2.872	0.0041
Instructor or assistant professor	169/196	86.20%	126/319	39.50%	10.408	<0.00001
Associate or full professor	13/196	6.60%	157/319	49.20%	-9.978	<0.00001
Pursue scholarship in medical education research	68/255	26.70%	92/498	18.50%	2.601	0.0093
Pursue scholarship in QA/QI	120/255	47.10%	191/498	38.40%	2.296	0.0214
Work with other hospitalists	146/260	56.20%	194/480	40.40%	4.101	<0.00001
Work with other trainees (fellows or residents)	190/260	73.10%	268/260	55.80%	4.611	<0.00001
Work with medical students	181/260	69.60%	254/480	52.90%	4.406	<0.00001
Compensation and salary perceptions						
Employees	248/265	93.60%	446/521	85.60%	3.29	0.001
Full/part-owners	7/265	2.60%	59/521	11.30%	-4.149	<0.00001
Provided health insurance	250/262	95.40%	459/513	89.50%	2.805	0.005
Provided short-term disability	195/262	74.40%	332/513	64.70%	2.741	0.0061
Very concerned about educational debt	64/265	24.20%	17/519	3.30%	9.084	<0.00001
No debt	87/265	32.80%	338/519	65.10%	-8.585	<0.00001
Believe their salary would be higher if hired by medicine	136/256	53.10%	168/495	33.90%	5.077	<0.00001
Responsible for their own practice costs	9/255	3.50%	66/494	13.40%	-4.247	<0.00001
Work for groups that see advantages to Med-Peds over categorical training	152/257	59.10%	241/496	48.60%	2.749	0.006
Career attitudes and plans						
Choose their current specialty if starting over	215/263	81.70%	372/521	71.40%	3.154	0.0016
Duty hour restrictions had a positive effect	135/256	52.70%	91/499	18.20%	9.798	<0.00001
Administration/leadership as a primary career path	20/250	8.00%	94/477	19.70%	-4.123	<0.00001
Clinician educator as a primary career path	45/250	18.00%	60/477	12.50%	1.975	0.0477
Clinician educator as a secondary career path	78/198	39.40%	86/312	27.60%	2.788	0.0053
Feel that hours are too long if reducing clinical time	25/74	33.80%	33/179	18.40%	2.642	0.0083

Overall, most reported being satisfied with their training (740/761, 97.2%) and would choose Med-Peds again (679/755, 89.9%). Most were pursuing a primary career as a staff clinician (460/730, 63%), and 186 of 743 (25%) planned to reduce their Med-Peds workload in the next five years (Table 6).

**Table 6:** Career attitudes and plans of Med-Peds physicians The numbers do not sum to 100% due to rounding and not all respondents answered every question resulting in variable denominators per question.

Career attitudes and plans	n	%
Satisfaction with training preparation		
Satisfied	740/761	97.20%
Neutral	10/761	1.30%
Unsatisfied	11/761	1.40%
Effect of duty hour restrictions on training		
Didn't train under	278/758	36.70%
Positive	226/758	29.80%
Neutral	222/758	29.30%
Negative	31/758	4.10%
Likely to choose Med-Peds again		
Likely	679/755	89.90%
Neutral	25/755	3.30%
Unlikely	51/755	6.80%
Agreement statements		
I am satisfied with my career as a physician	633/787	80.40%
If starting over, I would choose my current specialty	590/787	75.00%
Primary career path		
Staff clinician	460/730	63.00%
Administration/leadership	115/730	15.80%
Clinician educator	105/730	14.40%
Physician scientist	30/730	4.10%
Clinical expert	20/730	2.70%
Secondary career path		
Administration/leadership	195/512	38.10%
Clinician educator	165/512	32.20%
Staff clinician	82/512	16.00%
Clinical expert	52/512	10.20%
Physician scientist	18/512	3.50%
Five-year plan		
Reduce Med-Peds workload	186/743	25.00%
Move into a nonclinical role in medicine	91/743	12.20%
Retire from Med-Peds	69/743	9.30%
Pursue a career outside of medicine	34/743	4.60%
Pursue another specialty	16/743	2.20%
Primary reason for reducing clinical practice		
The hours are too long	58/254	22.80%
The practice is too stressful	34/254	13.40%
I am moving into a non-clinical position	46/254	18.10%
I like Med-Peds, but I have better opportunities outside the field	12/254	4.70%
I plan to retire	63/254	24.80%
I expect to lose my job due to downsizing or other market factors	0/254	0.00%
I dislike Med-Peds and hope for a better job offer outside the field	0/254	0.00%
Other (please specify)	41/254	16.10%

Trends in the workforce over time

When comparing early-career Med-Peds physicians (defined as being in the workforce for 10 years or less) and late-career physicians (defined as being in the workforce for over 10 years), there were statistically significant differences (Table 5). Early-career respondents were less likely to be working in solo practices, had spent less time with their current employer, and had fewer job changes. Early-career respondents were also less likely to be practicing part-time, more likely to be provided health insurance and short-term disability, had more debt concerns, believed duty hour restrictions had a positive effect on their training, and were less likely to retire in five years. However, there was no significant difference in the proportion of early-career versus late-career respondents wanting to reduce their workload in the next five years (55/255, 21.6% versus 130/488, 26.6%, respectively; p=0.128). Time with the current employer and the number of job changes were found to be linearly related to time since graduation, with a Pearson's coefficient of 0.06344 and 0.03608, respectively (p<0.001).

More surprisingly, early career physicians were working in urban communities at medical schools/hospitals and for institutions/groups that saw advantages to Med-Peds training. Although respondents primarily pursued a primary career as a staff clinician and a secondary career in administration/leadership, there had been a rise in those pursuing careers in clinical education as primary and secondary career paths. In addition to a rise in physician hospitalists, there was also a trend toward more physicians practicing both primary care and hospital medicine (26/258 versus 28/490; p=0.028). Fewer graduates were boarded only in pediatrics, which seems to have resulted in a proportional increase in double-boarded physicians. A larger proportion of Asian (60/288 versus 56/504; p=0.0002) and smaller proportion of White (202/288 versus 403/504; p=0.0017) physicians were entering the field of Med-Peds. There was also an increase in the percentage of Blacks/African Americans entering the field (18/288, 6.3% versus 17/504, 3.4%; p=0.0574). All other characteristics and attitudes of the Med-Peds workforce presented in Tables 1-4, 6 had no statistical difference (p-values >0.05) when comparing early with late-career physicians, including satisfaction in training, the likelihood of choosing Med-Peds again, and being satisfied with their career.

## Discussion

Clinical practice: primary care, hospitalist, and subspeciality

Several findings from this study have been consistent with the previous 2013 survey; however, much more has been revealed about the practice patterns, employment preferences, and concerns of the Med-Peds primary care workforce. While Med-Peds physicians remain actively engaged in primary care, a substantial number are choosing to practice hospital medicine, including a growing trend in combination hospital/primary care roles. However, when compared to internal medicine and pediatrics programs, the overall percentage of Med-Peds physicians practicing primary care is higher [7], suggesting a strong desire for Med-Peds graduates to remain in primary care. Still, hospital medicine appears to be a growing field with more Med-Peds physicians becoming members of the Society of Hospital Medicine (SHM). More recent evidence suggests this trend is plateauing around 24% of Med-Peds graduates [8], consistent with this study’s result of 27.4%. There also continue to be similar interests in subspecialty training when compared to the 2013 workforce survey. However, given this survey largely represents AAP members and recent evidence shows high attrition of Med-Peds graduates to internal medicine fellowships [8], this study is likely missing a large portion of subspecialist-related data. Other studies have noted as much as 40% of Med-Peds graduates pursue subspecialty careers [9], but graduate data does not account for several subspecialists identifying or dual practicing as primary care providers, such as infectious disease, adolescent medicine, geriatric, hospital, and academic specialists. Comparatively, this study shows 65.4% of respondents as practicing primary care.

Most Med-Peds physicians are engaged in direct patient care, with an even distribution of adult and pediatric patients (Table 2). This finding is similar to the age breakdown of patients in 2013 [2]. In the survey, 89.6% of Med-Peds primary care physicians saw patients across the age range of pediatric patients (less than 18 years of age) and adults (greater than 18 years of age). Despite practice changes endured in medicine over the last 10 years and the growing geriatric population, this has not affected the age ranges seen in this workforce. Many AAP members have also maintained membership with the American College of Physicians (ACP). This reaffirms the idea that most Med-Peds physicians practice both specialties in their careers [10]. This is vital as Med-Peds physicians are uniquely trained to care for complex pediatric patients into adulthood, reducing fragmentation or gaps in clinical services.

Patients with complex health needs, such as congenital heart disease and cystic fibrosis, are living longer [11,12] and the aging population highlights the growing demand for primary care services. Unfortunately, many of these shortages currently exist in rural settings [13,14], and this survey shows that Med-Peds physicians are inclined toward jobs in urban, university settings. This trend is likely influenced by the fact that only 2% of Medicare-funded residency positions occur in rural areas [15]. Although Congress recently created 1,000 new residency slots targeted toward training in rural and underserved areas, only 16.53 (4.13%) of the first 400 residency positions in 2023 went to pediatrics [15]. Strict Accreditation Council of Graduate Medical Education (ACGME) requirements also limit the existence of Med-Peds residency programs in areas with single institutional sponsorship of both internal medicine and pediatric programs [16]. While this survey was unable to determine if respondents preferred to work in a similar community compared to their training, AAMC data showed more than half of individuals practice in the state where they did residency, suggesting that they do not move far from their training grounds [17].

Adding to the limited number of Med-Peds training positions, this survey shows an equal proportion of early-career physicians wanting to reduce their workload compared to late-career physicians who are closer to retirement. That suggests a younger generation of physicians desiring a different work-life balance. This is consistent with AAMC’s 2022 survey findings that physicians expect to retire at an earlier age [3]. Therefore, a multi-specialty solution and thoughtfulness in the understanding of the younger generation's career goals is necessary to directly address the physician shortage and to provide seamless care to patients with complex medical needs.

Academia

One possible reason for Med-Peds physicians gravitating towards urban, university settings, is their propensity to maintain significant engagements in medical education. In addition to a large portion of the respondents holding a faculty appointment, there was a proportionally higher number of early-career physicians compared to late-career physicians pursuing medical education and QA/QI-related research activities. Early-career physicians have also surpassed late-career physicians in pursuing clinical education as a primary or secondary career, despite over 50% of late-career physicians working with trainees. These findings suggest a growing interest in academia and medical education in the Med-Peds workforce, as well as the development of a unique skill set due to the broad yet rigorous training requirements and the prevalence of Med-Peds training programs, particularly at larger academic institutions.

Diversity

Though not statistically significant, the percentage of Med-Peds women is higher than the results from 10 years ago. Over time, the percentage of women across the specialty has seemed to stabilize around 60% [4]. Not surprisingly, this data more closely resembles the gender differences seen in pediatrics presented by AAMC’s Physician Specialty Data Report 2022 [6]. This suggests that this survey’s respondents are more similar to pediatricians than internists. There is also a trend toward a more diverse workforce with a higher number of Asian physicians. However, racial differences do not seem to align with either pediatrics or internal medicine when compared to AAMC’s data, where rates of Asians in the workforce were slightly lower than AAMC’s Med-Peds results of 17.5% [6]. While the rise in the number of Black and African American physicians in the Med-Peds workforce survey has been modest, it is promising to see it is higher in Med-Peds than in medicine or pediatrics according to AAMC data (8.3% versus 6.7% and 6.5%, respectively) [6]. More concerning are the lower rates of Med-Peds physicians who identified as Hispanic or Latinx (4.8%) compared to medicine (6.3%) and pediatrics (7.8%) according to AAMC data [6] and mirrored in our survey results (2.4%). While ACGME has made efforts to emphasize and provide educational materials for the promotion of increasing diversity in medicine [18], these data suggest that more needs to be done in this area.

Training and certification

In 2024, there were 719 more positions offered in primary care compared to the previous match years; however, family medicine had 12% unfilled positions (636/5213), pediatrics had 8% (251/3078), and medicine had 5% (494/10261) [19]. Med-Peds had 100% filled positions (n=390), demonstrating continued interest in the specialty despite the concerns in other primary care fields [5]. While there are clear size differences, it remains that most Med-Peds physicians are satisfied with their career and training and would choose the same specialty again. This finding is consistent across Med-Peds physicians of all types: primary care physicians, hospitalists, and subspecialists. Concurrently, ACGME-imposed duty hour restrictions seem to positively affect the training of individuals who have completed Med-Peds residency. This, combined with the extremely high satisfaction with training preparation rates, suggests that Med-Peds residency programs provide sufficient training while adhering to duty hour restrictions despite an accelerated timeline for dual-specialty completion. 

While the majority of Med-Peds physicians continued to maintain dual certification in pediatrics and medicine, there was a reduction in the number of those solely boarded in pediatrics. These trends mirror the larger workforce concerns in pediatrics (shift towards part-time work, concern about compensation, transition to urban practice, and loss of rural access to care). This is further highlighted in the national trend moving away from pediatrics as seen by a steady decrease in pediatric residency and fellowship applicants over the past few years [20]. Med-Peds residency programs tend to draw in a competitive applicant pool, with 86.9% of the available positions in 2024 filled by US Doctors of Medicine (MDs) compared to 35% in internal medicine and 47.6% in pediatrics [5], contributing to the outcomes and career choices of the Med-Peds workforce. With the increase in double-boarded Med-Peds physicians and highly successful match rates, increasing the number of available Med-Peds training positions would likely serve to benefit the widening gap in the primary care workforce, with the consideration of supporting training programs or workforce opportunities within rural areas.

Compensation and debt

While there were several Med-Peds physicians without debt, there were growing concerns about educational debt between early-career versus late-career respondents. This is likely a result of rising medical education costs, which are increasing at 2.9% per year after adjusting for inflation [21]. About 70% of medical school graduates have educational debt in the mean amount of $206,000 in 2023 [22]. Medical school debt has wide-reaching effects on medical school graduates in terms of career choice, with those with more debt choosing higher compensation specialties. Despite these concerns, Med-Peds physicians showed confidence in their compensation and benefits, with less than 20% believing that Med-Peds training has a negative effect on salary. Interestingly, a large portion of Med-Peds physicians believed that their compensation would be higher if hired by internal medicine departments as opposed to pediatric departments. This is consistent with overall annual compensation rates of $312,526 for internists versus $259,579 for pediatricians reported by Doximity in 2024, with Med-Peds annual compensations falling around $273,472 [23]. Several pediatric subspecialties also lined the top 20 specialties with the lowest average annual compensation. In contrast, internal medicine subspecialties, such as cardiology, gastroenterology, and pulmonology, ranked among those with the highest annual compensation. Significant pay gaps by gender of around 23% also add to the inequitable compensation of pediatric professionals given a higher percentage of women in the field [23]. These differences in pay will continue to affect employee and employer decisions. While some employers will see a benefit of having Med-Peds physicians in pediatrician-sparse areas, Med-Peds employees may lean toward internal medicine for larger compensation packages. How these pressures will continue to affect the career decisions of new graduates and the Med-Peds workforce remains to be seen.

Limitations

There are several limitations to this study. The results of this survey primarily reflect the opinions of practicing Med-Peds members within the AAP, and despite the survey distribution through the MMS listserv of AMA members, the AAP’s listserv likely contained more up-to-date contact information of active members who had completed residency training. The survey also contained skip and display logic questions leading to variable response rates to each set of questions. However, likely due to the length of the questionnaire, there was also variation in the response rate to all available questions, leading to different denominators within question sets. It is also unclear what the motivations were of respondents answering some questions and not others. While most have maintained dual membership with the ACP, it is unclear if the reverse is true without an equivalent study distributed through the ACP. In 2022, the AAMC reported 6,088 Med-Peds physicians in the US [6]. This study likely represents 15% of the total workforce and is weighted toward primary care. Despite these limitations, it is important to recognize that little is published about the Med-Peds workforce nationally, and given the consistencies between this and the 2013 survey, these results are still generalizable to a large portion of the workforce.

## Conclusions

Med-Peds offers a unique and diverse training program for physicians, particularly those interested in primary care, medical education, and urban health. These survey findings provide valuable insights into the current state of the Med-Peds workforce and underscore the importance of this specialty in addressing evolving healthcare needs. The Med-Peds specialty continues to attract diverse talent, offering a rewarding career path with opportunities for growth and impact within the healthcare community. By addressing key areas such as workforce shortages, diversity, education costs, training and certification, and pay gaps, the Med-Peds workforce can continue to thrive and make meaningful contributions to the healthcare landscape.

## References

[1] Barr J (2024). The history of the Med-Peds program. http://publications.aap.org/pediatrics/resources/26198/The-History-of-the-Med-Peds-Program?autologincheck=redirected.

[2] Donnelly MJ, Thornton SC, Radabaugh CL, Friedland AR, Cross JT, Ruch-Ross HS (2015). Characteristics of the combined internal medicine-pediatrics workforce. Am J Med.

[3] (2024). The complexities of physician supply and demand: projections from 2021 to 2036. The Complexities of Physician Supply and Demand: Projections From 2021 to 2036.

[4] Sabety AH, Jena AB, Barnett ML (2021). Changes in health care use and outcomes after turnover in primary care. JAMA Intern Med.

[5] (2024). Results and data: 2024 main residency match. Results and Data: 2024 Main Residency Match®.

[6] (2025). US physician workforce data dashboard. http://www.aamc.org/data-reports/report/us-physician-workforce-data-dashboard.

[7] Moza R, Fish D, Peterson RJ (2022). Workforce characteristics of Med-Peds hospitalists. Cureus.

[8] Agrawal A, Aronica M, Kisielewski M, Doolittle B (2025). Career choices for graduates of combined medicine-pediatrics residency programs: a multi-year survey. J Gen Intern Med.

[9] Agrawal A, Wells D, Kisielewski M, Misra S, Doolittle B (2024). Subspecialty choices among medicine-pediatrics graduates: results from a four-year national program director survey. Cureus.

[10] Frohna JG, Melgar T, Mueller C, Borden S (2004). Internal medicine-pediatrics residency training: current program trends and outcomes. Acad Med.

[11] Pelosi C, Kauling RM, Cuypers JA (2023). Life expectancy and end-of-life communication in adult patients with congenital heart disease, 40-53 years after surgery. Eur Heart J Open.

[12] Singh H, Jani C, Marshall DC (2023). Cystic fibrosis-related mortality in the United States from 1999 to 2020: an observational analysis of time trends and disparities. Sci Rep.

[13] Lakhan SE, Laird C (2009). Addressing the primary care physician shortage in an evolving medical workforce. Int Arch Med.

[14] Butkus R, Rapp K, Cooney TG, Engel LS (2020). Envisioning a better US health care system for all: reducing barriers to care and addressing social determinants of health. Ann Intern Med.

[15] Rains J, Holmes GM, Pathak S, Hawes EM (2023). The distribution of additional residency slots to rural and underserved areas. JAMA.

[16] (2025). ACGME program requirements for graduate medical education in combined internal medicine-pediatrics. http://www.acgme.org/globalassets/pfassets/programrequirements/700_internalmedicinepediatrics_2023.pdf.

[17] (2025). Report on residents: executive summary. http://www.aamc.org/media/80361/download?attachment.

[18] Boatright D, London M, Soriano AJ (2023). Strategies and best practices to improve diversity, equity, and inclusion among US graduate medical education programs. JAMA Netw Open.

[19] (2024). Advanced data tables: 2024 main residency match. Advanced Data Tables: 2024 Main Residency Match®.

[20] (2024). 2024 ERAS applicant and application data. http://www.aamc.org/data-reports/data/eras-statistics-data.

[21] Mohareb AM, Brown TS (2023). Medical student debt and the US infectious diseases workforce. Clin Infect Dis.

[22] (2024). Medical student education: debt, costs, and loan repayment fact card for the class of 2023. Medical Student Education: Debt, Costs, and Loan Repayment Fact Card for the Class of 2023.

[23] (2024). Physician compensation report 2024. Physician Compensation Report.

